# Supplementary material for: Social disparities in the frequency and severity of triple-negative breast cancer at diagnosis in a university hospital in Paris, France: confronting race and ethnic blindness
Source: PLoS One. 2026 May 13;21(5):e0349041. doi: 10.1371/journal.pone.0349041 (PMC13170837; doi:10.1371/journal.pone.0349041)
Supplement: S1 File — (DOCX) [file pone.0349041.s001.docx]

**Supporting information 1. McGill Illness Narrative Interview (MINI)**

Groleau D, Young A, Kirmayer LJ. The McGill Illness Narrative Interview (MINI): an interview schedule to elicit meanings and modes of reasoning related to illness experience. *Transcult Psychiatry*. 2006 Dec;43(4):671-91. doi: 10.1177/1363461506070796.

**Section 1. INITIAL ILLNESS NARRATIVE**

1. When did you experience your health problem or difficulties (HP) for the first time? [*Substitute respondent’s terms for ‘HP’ in this and subsequent questions.*] [*Let the narrative go on as long as possible, with only simple prompting by asking*, ‘What happened then? And then?’]

2. We would like to know more about your experience. Could you tell us when you realized you had this (HP)?

3. Can you tell us what happened when you had your (HP)?

4. Did something else happen? [*Repeat as needed to draw out contiguous experiences* *and events.*]

5. If you went to see a helper or healer of any kind, tell us about your visit and what happened afterwards.

6. If you went to see a doctor, tell us about your visit to the doctor/hospitalization and about what happened afterwards.

6.1 Did you have any tests or treatments for your (HP)? [*The relevance of this question* *depends on the type of health problem.*]

**Section 2. PROTOTYPE NARRATIVE**

7. In the past, have you ever had a health problem that you consider similar to your current (HP)? [If answer to #7 is Yes, then ask Q.8]

8. In what way is that past health problem similar to or different from your current (HP)?

9. Did a person in your family ever experience a health problem similar to yours? [If answer to #9 is Yes, then ask Q.10]

10. In what ways do you consider your (HP) to be similar to or different from this other person’s health problem?

11. Did a person in your social environment (friends or work) experience a health problem similar to yours? [If answer to #11 is Yes, then ask Q.12]

12. In what ways do you consider your (HP) to be similar to or different from this other person’s health problem?

13. Have you ever seen, read or heard on television, radio, in a magazine, a book or on the Internet of a person who had the same health problem as you? [If answer to #13 is

Yes, then ask Q.14]

14. In what ways is that person’s problem similar to or different from yours?

**Section 3. EXPLANATORY MODEL NARRATIVE**

15. Do you have another term or expression that describes your (HP)?

16. According to you, what caused your (HP)? [List primary cause(s).]

16.1 Are there any other causes that you think played a role? [List secondary causes.]

17. Why did your (HP) start when it did?

18. What happened inside your body that could explain your (HP)?

19. Is there something happening in your family, at work or in your social life that could explain your health problem? [If answer to #19 is Yes, then ask Q.20]

20. Can you tell me how that explains your health problem?

21. Have you considered that you might have *[INTRODUCE POPULAR SYMPTOM*

*OR ILLNESS LABEL]*?

22. What does *[POPULAR LABEL]* mean to you?

23. What usually happens to people who have *[POPULAR LABEL]?*

24. What is the best treatment for people who have *[POPULAR LABEL]?*

25. How do other people react to someone who has *[POPULAR LABEL]?*

26. Who do you know who has had *[POPULAR LABEL]*?

27. In what ways is your (HP) similar to or different from that person’s health problem?

28. Is your (HP) somehow linked or related to specific events that occurred in your life?

29. Can you tell me more about those events and how they are linked to your (HP)?

**Section 4. SERVICES AND RESPONSE TO TREATMENT**

30. During your visit to the doctor (healer) for your HP, what did your doctor (healer)

tell you that your problem was?

31. Did your doctor (healer) give you any treatment, medicine or recommendations to follow? [List all]

32. How are you dealing with each of these recommendations? [*Repeat Q. 33 to Q. 36 as needed for every recommendation, medicine and treatment listed.*]

33. Are you able to follow that treatment (or recommendation or medicine)?

34. What made that treatment work well?

35. What made that treatment difficult to follow or work poorly?

36. What treatments did you expect to receive for your (HP) that you did not receive?

37. What other therapy, treatment, help or care have you sought out?

38. What other therapy, treatment, help or care would you like to receive?

**Section 5. IMPACT ON LIFE**

39. How has your (HP) changed the way you live?

40. How has your (HP) changed the way you feel or think about yourself?

41. How has your (HP) changed the way you look at life in general?

42. How has your (HP) changed the way that others look at you?

43. What has helped you through this period in your life?

44. How have your family or friends helped you through this difficult period of your life?

45. How has your spiritual life, faith or religious practice helped you go through this difficult period of your life?

46. Is there anything else you would like to add?
