# Supplementary material for: Social disparities in the frequency and severity of triple-negative breast cancer at diagnosis in a university hospital in Paris, France: confronting race and ethnic blindness
Source: PLoS One. 2026 May 13;21(5):e0349041. doi: 10.1371/journal.pone.0349041 (PMC13170837; doi:10.1371/journal.pone.0349041)
Supplement: S2 File — (DOCX) [file pone.0349041.s002.docx]

**Supporting information 2. Sensitivity analysis for missing tumor markers data.**

Due to missing data for one or more tumor markers, triple-negative status was unknown for 56 women: 10 of 177 SSA women (5.6%), 14 of 153 MAG (9.2%), and 3 of 336 FRA (0.9%), p<0.001.

**Table S1. Comparison of patients without and with missing data for one or more tumor markers, by origin.**

|  |  | SS Africa | | Maghreb | | France | |
| --- | --- | --- | --- | --- | --- | --- | --- |
|  |  | No MV  n=167 | MV n=10 | No MV  n=139 | MV  n=14 | No MV  n = 333 | MV  n=3 |
| Age: average (sd) | | 54.1 (11.9) |  | 60.7 (12.7) |  | 65.2 (12.9) |  |
|  | | % | % | % | % | % | % |
| Health insurance status | |  |  |  |  |  |  |
| SS | | 35.9 | 10.0 | 99.0 | 100.0 | 66.9 | 57.1 |
| CMUc | | 8.4 | 20.0 | 0.7 | 0.0 | 9.4 | 7.1 |
| AME | | 35.9 | 50.0^1^ | 0.0 | 0.0 | 11.5 | 28.6^2^ |
| Paid care | | 19.8 | 20.0 | 0.3 | 0.0 | 12.2 | 7.1 |
| Combined Occupation category | | | |  |  |  |  |
| Other | | 38.3 | 30.0 | 41.7 | 28.6 | 55.3 | 30.0 |
| Not in the labor force or low-skilled occupation* | | 61.7 | 70.0^3^ | 58.3 | 71.4^4^ | 44.7 | 70.0^5^ |

^1^p=0.50 (AME vs others); ^2^p=0.09 (AME vs others); ^3^p=0.74; ^4^p=0.40; ^5^p=0.26 (all using Fisher's exact test).

A sensitivity analysis was performed, assuming all missing values were positive or negative, respectively.

**Table S2. Sensitivity analysis for the frequency of TNBC.**

|  | Sub-Saharan Africa | Maghreb | France |
| --- | --- | --- | --- |
| **Results reported*** | n=167 | n=142 | n=333 |
| TNBC | 38.3% | 16.5% | 12.2% |
| 95%CI | [31.2%-45.8%] | [11.1%-23.4%] | [8.9%-16.2%] |
| **Sensitivity analysis** | n=177 | n=153 | n=336 |
| *if all missing values for triple negative status = "yes"* | | |  |
| TNBC | 41.8% | 24.2% | 20.5% |
| 95%CI | [34.7%-49.2%] | [17.9%-31.4%] | [16.5%-25.1%] |
| *if all missing values for triple negative status = "no"* | | |  |
| TNBC | 36.2% | 15.0% | 11.0% |
| 95%CI | [29.4%-43.4%] | [10.0%-21.3%] | [8.0%-14.7%] |

**referring to Figure 1 of the main article*

**Table S3. Factors associated with stage T≥3 (multivariate analysis), if all missing values for triple negative status = "yes"**

|  |  | aOR | 95% CI | p |
| --- | --- | --- | --- | --- |
| Age (years) |  | 0.99 | [0.97-1.0] | 0.07 |
| Triple negative | No | Ref |  | 0.98 |
|  | Yes | 1.00 | [0.66-1.55] |  |
| Region of birth | France | Ref |  | <0.001 |
|  | Maghreb | 1.56 | [0.91-2.65] |  |
|  | Sub-Saharan Africa | 3.25 | [1.86-5.67] |  |
| Health insurance status | SS | Ref |  | 0.68 |
|  | CMUc | 1.10 | [0.47-2.60] |  |
|  | AME | 1.00 | [0.54-1.84] |  |

**Table S4. Factors associated with stage T≥3 (multivariate analysis), if all missing values for triple negative status = "no"**

|  |  | aOR | 95% CI | p |
| --- | --- | --- | --- | --- |
| Age (years) |  | 0.99 | [0.97-1.00] | 0.09 |
| Triple negative | No | Ref |  | 0.29 |
|  | Yes | 1.28 | [0.81-2.03] |  |
| Region of birth | France | Ref |  | <0.001 |
|  | Maghreb | 1.75 | [1.00-3.03] |  |
|  | Sub-Saharan Africa | 3.17 | [1.78-5.65] |  |
| Health insurance status | SS | Ref |  | 0.98 |
|  | CMUc | 1.04 | [0.44-2.44] |  |
|  | AME | 0.95 | [0.51-1.76] |  |

**Table S5. Sensitivity analysis for the frequency of stage T≥3 tumors by country of birth according to triple negative status.**

|  | Sub-Saharan Africa | | France | |
| --- | --- | --- | --- | --- |
|  | TNBC | Other | TNBC | Other |
| **Results reported** | n=167 | | n=333 | |
| T>=3 | 46.9% | 36.9% | 27.0% | 12.7% |
| 95%CI | [35.0%-59.0%] | [28.0%-46.5%] | [14.8%-42.7%] | [9.1%-17.1%] |
| **Sensitivity analysis** | n=177 | | n=336 | |
| *if all missing values for triple negative status = "yes"* | | |  |  |
| T>=3 | 43.2% | 36.9% | 15.9% | 12.7% |
| 95%CI | [32.4%-54.6%] | [28.0%-46.5%] | [8.8%-25.9%] | [9.1%-17.1%] |
| *if all missing values for triple negative status = "no"* | | |  |  |
| T>=3 | 46.9% | 35.4% | 27.0% | 11.7% |
| 95%CI | [35.0%-59.0%] | [27.0%-44.5%] | [14.8%-42.7%] | [8.4%-15.7%] |

**referring to Figure 3 of the main article*
