# Supplementary material for: Social disparities in the frequency and severity of triple-negative breast cancer at diagnosis in a university hospital in Paris, France: confronting race and ethnic blindness
Source: PLoS One. 2026 May 13;21(5):e0349041. doi: 10.1371/journal.pone.0349041 (PMC13170837; doi:10.1371/journal.pone.0349041)
Supplement: S3 File — (DOCX) [file pone.0349041.s003.docx]

**Supporting information 3. Frequency of TNBC and factors associated among local residents only (excluding the 54 patients coming for treatment from abroad)**

**Table S1. Frequency of TNBC by country of birth (% and 95% CI)..**

|  | Sub-Saharan Africa | Maghreb | France |
| --- | --- | --- | --- |
| **Results reported*** | n=167 | n=142 | n=333 |
| TNBC | 38.3% | 16.5% | 12.2% |
| 95%CI | [31.2%-45.8%] | [11.1%-23.4%] | [8.9%-16.2%] |
| **Local residents only** | n=142 | n=135 | n=332 |
| TNBC | 38.1% | 17.2% | 12.2% |
| 95%CI | [30.2%-46.5%] | [11.3%-24.6%] | [8.9%-16.3%] |

**referring to Figure 1 of the main article, including patients from abroad*

**Table S2. Factors associated with TNBC (multivariate analysis): Model 1**

|  | Results reported^1^ | | | Local residents only | | |
| --- | --- | --- | --- | --- | --- | --- |
|  | aOR | 95% CI | p | aOR | 95% CI | p |
| Age | 0.99 | [0.97-1.00] | 0.09 | 0.98 | [0.97-1.00] | 0.09 |
| Region of birth |  |  | <0.001 |  |  | <0.001 |
| France | ref |  |  | ref |  |  |
| Maghreb | 1.34 | [0.76-2.37] |  | 1.42 | [0.79-2.55] |  |
| Sub-Saharan Africa | 3.86 | [2.37-6.31] |  | 3.71 | [2.20-6.24] |  |

^1^ *referring to Table 3 of the main article, including patients from abroad*

**Table S3. Factors associated with TNBC (multivariate analysis): Model 2**

|  | Results reported^1^ | | | Local residents only | | |
| --- | --- | --- | --- | --- | --- | --- |
|  | aOR | 95% CI | p | aOR | 95% CI | p |
| Age | 0.99 | [0.97-1.00] | 0.09 | 0.99 | [0.97-1.00] | 0.09 |
| Region of birth |  |  | 0.01 |  |  | 0.01 |
| France | ref |  |  | ref |  |  |
| Maghreb | 1.08 | [0.58-1.99] |  | 1.15 | [0.62-2.14] |  |
| Sub-Saharan Africa | 2.72 | [1.49-4.96] |  | 2.48 | [1.33-4.61] |  |
| Health insurance status |  |  | 0.02 |  |  | 0.04 |
| SS | ref |  |  | ref |  |  |
| CMUc | 1.55 | [0.63-3-84] |  | 1.57 | [0.63-3-86] |  |
| AME | 1.85 | [1.01-3.50] |  | 1.92 | [1.01-3.65] |  |
| Combined Occupation category |  |  | 0.049 |  |  | 0.04 |
| Other | ref |  |  | ref |  |  |
| Not in the labor force or low-skilled occupation* | 1.56 | [1.01-2.42] |  | 1.62 | [1.02-2.58] |  |

^1^ *referring to Table 3 of the main article, including patients from abroad*

*blue collar or lower white collar

**Table S4. Factors associated with TNBC (multivariate analysis): Model 1, further adjusted on tumor stage**

|  | Results reported^1^ | | | Local residents only | | |
| --- | --- | --- | --- | --- | --- | --- |
|  | aOR | 95% CI | p | aOR | 95% CI | p |
| Age | 0.99 | [0.97-1.00] | 0.09 | 0.99 | [0.97-1.00] | 0.09 |
| Region of birth |  |  | <0.001 |  |  | <0.001 |
| France | ref |  |  | ref |  |  |
| Maghreb | 1.34 | [0.76-2.37] |  | 1.37 | [0.76-2.47] |  |
| Sub-Saharan Africa | 3.86 | [2.37-6.31] |  | 3.36 | [1.98-5.71] |  |
| Tumor stage T≥3 |  |  |  | 1.68 | [1.03-2.75] | 0.04 |

^1^ *referring to Table 3 of the main article, including patients from abroad*

**Table S5. Factors associated with TNBC (multivariate analysis): Model 2, further adjusted on tumor stage**

|  | Results reported^1^ | | | Local residents only | | |
| --- | --- | --- | --- | --- | --- | --- |
|  | aOR | 95% CI | p | aOR | 95% CI | p |
| Age | 0.99 | [0.97-1.00] | 0.09 | 0.99 | [0.97-1.00] | 0.20 |
| Region of birth |  |  | 0.01 |  |  | 0.025 |
| France | ref |  |  | ref |  |  |
| Maghreb | 1.08 | [0.58-1.99] |  | 1.12 | [0.60-2.09] |  |
| Sub-Saharan Africa | 2.72 | [1.49-4.96] |  | 2.25 | [1.20-4.23] |  |
| Health insurance status |  |  | 0.02 |  |  | 0.04 |
| SS | ref |  |  | ref |  |  |
| CMUc | 1.55 | [0.63-3-84] |  | 1.53 | [0.61-3-79] |  |
| AME | 1.85 | [1.01-3.50] |  | 1.95 | [1.02-3.72] |  |
| Combined Occupation category |  |  | 0.049 |  |  | 0.044 |
| Other | ref |  |  | ref |  |  |
| Not in the labor force or low-skilled occupation* | 1.56 | [1.01-2.42] |  | 1.61 | [1.01-2.57] |  |
| Tumor stage T≥3 |  |  |  | 1.68 | [1.02-2.75] | 0.04 |

^1^ *referring to Table 3 of the main article, including patients from abroad*

*blue collar or lower white collar
